# Beaked Whales Respond to Simulated and Actual Navy Sonar

**DOI:** 10.1371/journal.pone.0017009

**Published:** 2011-03-14

**Authors:** Peter L. Tyack, Walter M. X. Zimmer, David Moretti, Brandon L. Southall, Diane E. Claridge, John W. Durban, Christopher W. Clark, Angela D'Amico, Nancy DiMarzio, Susan Jarvis, Elena McCarthy, Ronald Morrissey, Jessica Ward, Ian L. Boyd

**Affiliations:** 1 Biology Department, Woods Hole Oceanographic Institution, Woods Hole, Massachusetts, United States of America; 2 North Atlantic Treaty Organisation Undersea Research Centre, La Spezia, Italy; 3 Naval Undersea Warfare Center Division, Newport, Rhode Island, United States of America; 4 Southall Environmental Associates, Aptos, California, United States of America; 5 Long Marine Laboratory, University of California Santa Cruz, Santa Cruz, California, United States of America; 6 Bahamas Marine Mammal Research Organisation, Marsh Harbour, Abaco, Bahamas; 7 Protected Resources Division, Southwest Fisheries Science Center, National Marine Fisheries Service, National Oceanic and Atmospheric Administration, La Jolla, California, United States of America; 8 Bioacoustics Research Program, Cornell Lab of Ornithology, Cornell University, Ithaca, New York, United States of America; 9 Space and Naval Warfare Systems Center Pacific, San Diego, California, United States of America; 10 Sea Mammal Research Unit, Scottish Oceans Institute, University of St. Andrews, Fife, Scotland, United Kingdom; National Institute of Water & Atmospheric Research, New Zealand

## Abstract

Beaked whales have mass stranded during some naval sonar exercises, but the cause is unknown. They are difficult to sight but can reliably be detected by listening for echolocation clicks produced during deep foraging dives. Listening for these clicks, we documented Blainville's beaked whales, *Mesoplodon densirostris*, in a naval underwater range where sonars are in regular use near Andros Island, Bahamas. An array of bottom-mounted hydrophones can detect beaked whales when they click anywhere within the range. We used two complementary methods to investigate behavioral responses of beaked whales to sonar: an opportunistic approach that monitored whale responses to multi-day naval exercises involving tactical mid-frequency sonars, and an experimental approach using playbacks of simulated sonar and control sounds to whales tagged with a device that records sound, movement, and orientation. Here we show that in both exposure conditions beaked whales stopped echolocating during deep foraging dives and moved away. During actual sonar exercises, beaked whales were primarily detected near the periphery of the range, on average 16 km away from the sonar transmissions. Once the exercise stopped, beaked whales gradually filled in the center of the range over 2–3 days. A satellite tagged whale moved outside the range during an exercise, returning over 2–3 days post-exercise. The experimental approach used tags to measure acoustic exposure and behavioral reactions of beaked whales to one controlled exposure each of simulated military sonar, killer whale calls, and band-limited noise. The beaked whales reacted to these three sound playbacks at sound pressure levels below 142 dB re 1 µPa by stopping echolocation followed by unusually long and slow ascents from their foraging dives. The combined results indicate similar disruption of foraging behavior and avoidance by beaked whales in the two different contexts, at exposures well below those used by regulators to define disturbance.

## Introduction

Over the past 20 years, there has been increasing concern that noise from human activities may affect wildlife. Recent work has identified impacts of anthropogenic sound on terrestrial birds [1] and anurans [2]. Sound propagates underwater with much less loss than in air, so sounds produced underwater may impact animals over greater ranges than sounds produced in air. The first alarms about the effect of anthropogenic sound on wildlife concerned marine life [3], including fish [4] and marine mammals [5], [6]. Concern ranges from effects such as shipping noise reducing the range over which whales can communicate [5] to injury from intense sound [6]. Regulations designed to protect marine mammals against injury from loud sounds are based upon studies that test how much sound exposure is required to cause temporary decreases in the sensitivity of hearing. These studies suggest that risk of injury from exposure to loud sound sources such as military sonars should be limited to tens of meters [6]. It was therefore surprising when evidence mounted that atypical mass strandings of beaked whales (family Ziphiidae) have coincided with some naval exercises involving mid-frequency active (MFA) sonars, which operate in the 3–8 kHz frequency band [7]–[9]. These strandings are the only known cases where exposure to anthropogenic underwater sound has been demonstrated to lead to the death of marine mammals [10].

Two species of beaked whale are most commonly involved in sonar-related mass strandings: Cuvier's (*Ziphius cavirostris*) and Blainville's (*Mesoplodon densirostris*) beaked whales [8]. These animals regularly dive to >1000 m and for >1 h, making them some of the most extreme divers among air-breathing animals [11]. Beaked whales are difficult to sight during their infrequent surfacings but they can be detected by listening for the echolocation clicks they produce during deep foraging dives [11], [12]. It is impossible to reconstruct the acoustic exposure of beaked whales that stranded during sonar exercises, because the location where they first heard the sonar is unknown, but it is very unlikely that they could have been exposed to sound levels thought to pose a risk of direct physical injury if the risk is limited to the predicted range of tens of meters from the ship. There is a growing consensus that exposure to military sonar may trigger a behavioral reaction in beaked whales that may lead to lethal stranding, and that the behavioral reaction may also lead to injuries related to bubble growth through decompression as the whales alter their dive behavior [7]. This paper addresses the critical questions of how to safely determine the sound exposures that cause beaked whales to initiate behavioral reactions to sonar, and of how to define these reactions.

One puzzle about sonar related strandings of beaked whales is that mid-frequency sonars operate at frequencies well below those at which beaked whales hear best [13], [14], and well below the frequency range of their own vocalizations, which have a center frequency of about 40 kHz [15]. The logic used for many environmental risk assessments would consider MFA sonars to pose a low risk of acoustic disturbance to beaked whales based on this frequency mismatch alone (see [6] for a review of frequency-selective weighting of sound stimuli based upon hearing sensitivity). Yet the evidence is clear that MFA sonar can trigger a response leading to lethal strandings. There can be significant energy in harmonics of the fundamental frequency of MFA sonars, and it is possible that these harmonics may be involved in the response. Another hypothesis for why these tonal mid-frequency signals trigger such a strong response notes their acoustic similarity to the calls of killer whales [16]. Each of these hypotheses suggests different approaches for reducing the risk to beaked whales from exposure to sonar. Resolving these issues has importance well beyond beaked whales and sonar, as they call into question some of the basic assumptions used to predict impacts of anthropogenic sounds on wildlife.

The importance of understanding behavioral responses of beaked whales to sonar sounds and the extreme difficulty in studying them calls for a strategic integration of diverse approaches using advanced sensing and tracking technologies, each operating on different spatial and temporal scales. This paper approaches the problem of studying the behavioral responses of beaked whales to sonars using these different methods in controlled experiments and observational studies. The experiments are so difficult to conduct that they involve a small sample size that is supplemented by a much larger sample in the observational studies of responses to actual sonar exercises. For the controlled exposure experiments, we selected three different stimuli: a MFA sonar signal with minimal energy in harmonics, calls of killer whales filtered to a bandwidth similar to that of MFA sonar, and a pseudorandom noise (PRN) stimulus with the same overall timing and bandwidth as the MFA stimulus but with an acoustic fine-structure very different from MFA or killer whale calls. The hypothesis noted above that beaked whales may treat MFA sonar as indicating the possible presence of a killer whale would predict similar responses at similar exposure levels for MFA and killer whale stimuli. Alternatively, if the whales are simply responding to the broader timing and frequency bands of sound, as is usually predicted in environmental assessments [6], then they would be predicted to respond similarly to MFA and PRN stimuli. The research described here succeeded in developing innovative methods to define responses of beaked whales to actual sonar exercises, and to measure sound exposures leading to initial reactions of tagged whales to experimental exposures to sonar and other sounds.

## Results

### Studying responses of beaked whales to sonar exercises

We used satellite tags and newly developed acoustic monitoring methods to follow changes in the location and foraging behavior of Blainville's beaked whales, *Mesoplodon densirostris*, before, during, and after naval sonar exercises. This observational study took advantage of acoustic tracking capabilities of a US Navy underwater testing range that hosts naval sonar exercises. This range, called the Atlantic Undersea Test and Evaluation Center (AUTEC), in the Tongue of the Ocean near Andros Island in the Bahamas, has an array of bottom-mounted hydrophones designed to track vessels that produce sounds within the range. The capabilities of the AUTEC range have been adapted to monitor the sounds of marine mammals, including clicks of beaked whales [17]. Before acoustic monitoring, beaked whales were thought to be rare at the AUTEC range, but echolocation clicks from foraging groups of Blainville's beaked whales are routinely detected over several hydrophones for about half an hour at a time [17]. Acoustic monitoring during 2005 suggested the presence of about 25 whales on the 1124 km^2^ range when no sonar was being used [18].

### Passive acoustic monitoring of beaked whales before, during, and after sonar exercises

The length of time over which a group of clicking beaked whales is detected acoustically during synchronized foraging dives is called a group clicking period (GCP). We studied the location and duration of GCPs before, during, and after two naval sonar exercises in 2007 and 2008 that each involved three ships using mid-frequency active sonars (MFA) including AN/SQS-56 or AN/SQS-53C sonars whose use has been associated with beaked whale strandings [7], [8]. These exercises also involved a variety of other sound sources including dipping sonars deployed from helicopters, underwater acoustic communication systems, pingers used for tracking on the range, sonar countermeasures, sonobuoys, and ship propulsion noise [19]. The period before sonar exposure was defined as Pre-exposure, that during the sonar exercise as Exposure. The period after sonar exposure was treated slightly differently in 2007, when the entire post-exposure period of 23 h was defined as a Recovery phase, compared to 2008 when 108 h of post-exposure measurements were subdivided into a 65 h Recovery phase and a 43 h Post-recovery phase.

**Figure 1:**
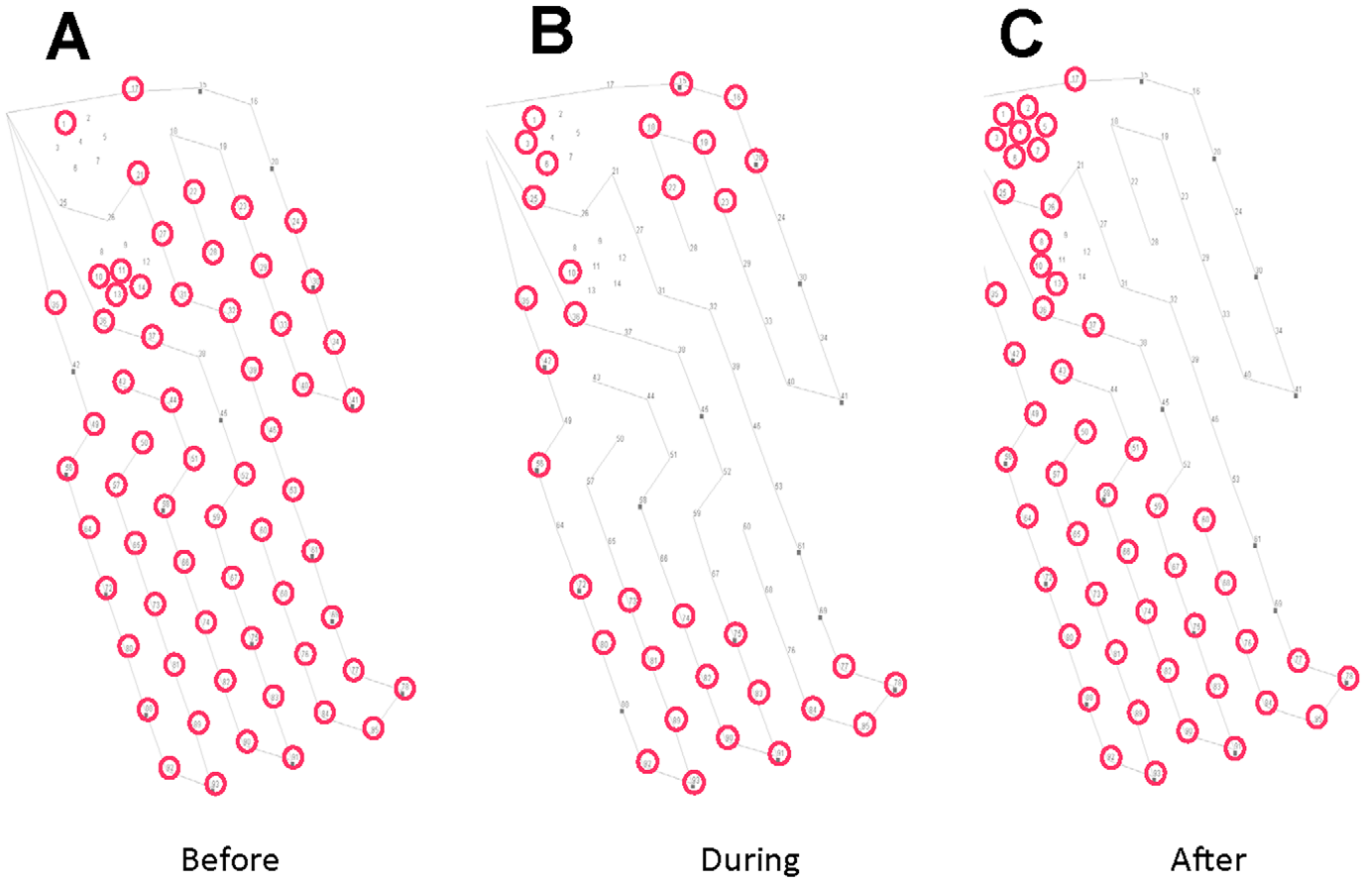
Maps of the AUTEC hydrophone array indicating presence of beaked whales. Hydrophones that detected Blainville's beaked whale clicks are circled in red. (A) Hydrophones that detected whales during 20 h before a sonar exercise from May 2007. (B) Hydrophones that detected whales over 23 h during the exercise. (C) Hydrophones that detected whales during 22 h after the exercise stopped. Fewer hydrophones detected beaked whales during the sonar exercise compared to before and after, with no detections in the center of the range during sonar operations.

Fig. 1A–C shows maps of the AUTEC hydrophone array, with hydrophones circled in red if they detected clicks from one or more Blainville's beaked whales on the range for 20 h before, 23 h during, and 22 h after active sonar operations in May 2007. As is common during baseline conditions, beaked whales were detected throughout the range before sonar operations began (Fig. 1A). During the sonar exercise, involving bouts of sonar transmission lasting up to 6 h, no detections were recorded on hydrophones in the center of the range where most sonar activities were concentrated (Fig. 1B). After the sonar exercise, detections of GCPs gradually spread from the periphery to the center of the range (Fig. 1C).

Table 1 tallies the number and rate of group clicking periods (GCPs) for periods before, during, and after sonar exercises at AUTEC during 2007 and 2008. In order to quantify whether detections occurred in the center or periphery of the range, we defined “Edge” hydrophones as the outermost phones at the perimeter of the range. Table 1 lists the percentage of group clicking periods detected on hydrophones at the edge of the range during each phase of the sonar exercises.

**Table 1:** Effects of sonar activity at AUTEC on the location and acoustic behavior of beaked whales.

	Year							
	2007				2008			
	Duration (h)	Number of GCPs	GCP Rate (number/h)	% of GCPs on edge hydro-phones	Duration (h)	Number of GCPs	GCP Rate (number/h)	% of GCPs on edge hydro-phones
Before	17	63	3.71	35	65	263	4.05	36
During	75	82	1.09	68	68	93	1.37	48
After (Recovery)	23	51	2.22	25	65	97	1.49	23
After (Post-Recovery)	No data	No data	No data	No data	43	265	6.16	25

“Duration” is the duration of the sample period. A group clicking period (GCP) is a spatially and temporally distinct set of echolocation clicks that indicates a group of beaked whales foraging during a deep dive. “Edge” hydrophones were the outermost phones at the perimeter of the range.

**Figure 2:**
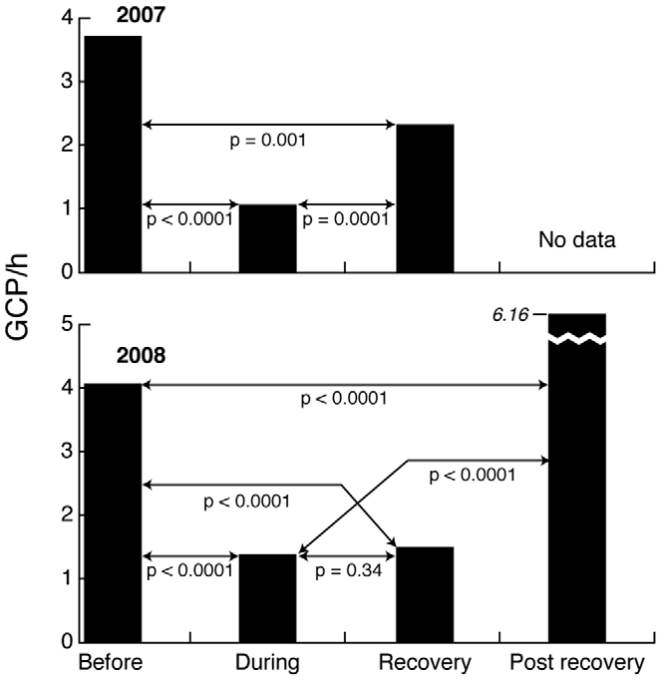
Variation in Group Clicking Periods (GCP) of Blainville's beaked whales exposed to sonar exercises. GCPs were defined by detections of beaked whale clicks within a cluster of hydrophones, representing synchronized deep foraging dives of a group of beaked whales.

A Monte Carlo simulation showed that there was a significant (p<0.0001) reduction in the rate of GCPs observed on the AUTEC range during sonar operations compared with before operations in both years (Fig. 2). Following sonar operations, GCP rates had not fully recovered to the pre-exposure levels after 23 h in 2007 and after 65 h in 2008 (Fig. 2). During the post-recovery period in 2008, 65–108 h after exposure ended, the GCP rate was significantly greater than the pre-exposure rate (Fig. 2). These combined observations suggest that the whales took three days to recover from exposure and then may have increased their foraging.

The received sound exposure at each beaked whale group that was detected clicking during sonar operations could be estimated using the location of the group relative to the sound source, the nominal source level for each kind of sonar used in the exercise, times of sonar transmissions and an established sound propagation model [20]. Table 2 lists exposure data for all beaked whales whose clicks were detected during sonar exercises in 2007 and 2008, where the clicks could be localized sufficiently to estimate location. These animals were located between 2.2–28.9 km away from naval ships transmitting sonar signals. Of the 17 GCPs analyzed during sonar transmissions, the estimated received levels ranged from 101 to 157 dB re 1 µPa, with an average received level of 128 dB re 1 µPa for animals that continued to forage during sonar exposure.

**Table 2:** Summary of received levels of sonar sound on Blainville's beaked whales monitored during military sonar operations in 2007 and 2008.

Sonar Type	Frequency (kHz)	Range (km)	Received Level (dB re 1 µPa) (rms)
53C	3.5	19.0	127
56	8	21.4	114
53C	3.5	18.2	133
56	8	25.2	112
53C	3.5	14.7	133
56	8	21.1	118
56	8	12.2	117
Other	4.5	24.3	117
Other	4.5	3.4	154
Other	4.5	2.2	157
56	8	7.0	143
56	8	12.6	126
56	8	11.4	129
56	8	7.6	141
56	8	28.9	101
*53C	3.5	18.1	128
*56	8	25.8	118

The last two entries are marked with an asterisk because they represent the same GCP, which was simultaneously exposed to two different sonars.

We compared the proportion of GCPs on edge vs center hydrophones in the expectation that if beaked whales moved off the range when sonars were active, then there would be more detections of group clicking periods on the edge of the range compared to the center during that active sonar period. The percentage of GCPs detected on edge hydrophones was higher during the sonar exercises than either before or after, suggesting that animals moved off range during sonar exercises and slowly returned afterwards. However, acoustic monitors could not directly discriminate whether whales in the center of the range just silenced or whether they moved out of the center of the range.

### Satellite tag tracking of a beaked whale before, during, and after a sonar exercise

**Figure 3:**
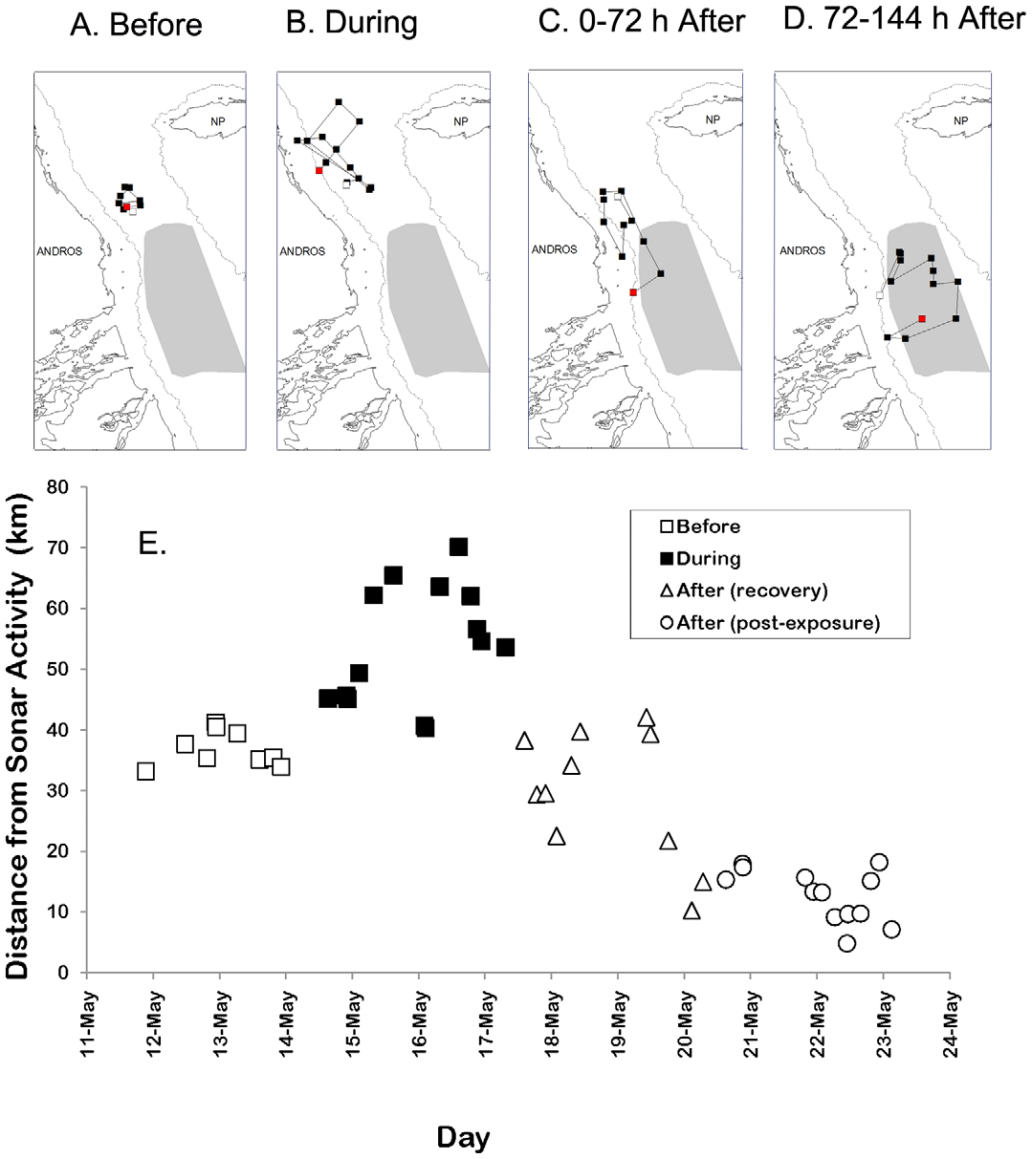
Locations of a Blainville's beaked whale satellite tagged before a naval sonar exercise in the AUTEC range in May 2009. For each segment of the track in subplots A–D, the start is plotted with a white square and the end is marked with a red square. The shaded area indicates the extent of the AUTEC range hydrophone array. (A) Locations recorded 72 h before a sonar exercise started on the AUTEC range. (B) Locations recorded during the 72 h sonar exercise. (C) Locations recorded 72 h after the exercise ended. (D) Locations recorded between 72 and 144 h after the exercise ended. (E) Distance from each location to the center of the AUTEC range as a function of date.

To track movements of an individual whale during a sonar exercise, a satellite transmitter tag [21] was attached to an adult male Blainville's beaked whale within the AUTEC range on 7 May 2009. During the 72 h before the sonar exercise started, the mean distance from whale to the center of the AUTEC range was 37 km±3 (s.d.) (Fig. 3A, E). During the 72 h sonar exercise, the whale moved several tens of km farther away (mean distance 54 km±10 (s.d.), Fig. 3B, E). The received sound levels at the tagged whale during sonar exposure were estimated using the same method as used with the GCPs, with the highest received level estimated at 146 re 1 µPa. The tagged whale slowly returned for several days after the exercise stopped (mean distance 29 km±11 (s.d.) from 0–72 h after the exercise stopped, Fig. 3C, E; and 13 km±4 (s.d.) from 72–144 h after the exercise stopped, Fig. 3D, E). The tag data support the interpretation that when GCPs are not detected in the center of the range, the whales have not just silenced, but have also moved away, and that it takes about 3 days for them to return.

### Responses of tagged beaked whales to experimental playback of sonar and other sounds

Observations of responses of whales to actual exercises can help frame the spatial and temporal scales of response, and with appropriate models and environmental data, one can estimate exposure levels associated with the response. However, the opportunistic studies described above were restricted to limited observations of movement and vocal behavior, and they measure responses to a complex mixture of stimuli. A tag has been developed to record acoustic exposure at an animal along with detailed continuous measurements of subtle details of vocal and movement behavior [22]. This tag was used to conduct field playback experiments on the AUTEC range, exposing tagged Blainville's beaked whales to three stimuli designed to test different hypotheses about the acoustic features to which the whales are responding: simulated mid-frequency sonar signals (MFA), a pseudo-random noise signal (PRN) with timing and bandwidth similar to the sonar, and calls of killer whales (*Orcinus orca*) [23]. The experimental design called for starting exposure at a low level, gradually increasing the level until a response was observed. This dose escalation was designed to minimize overall exposure while defining the lowest level at which the animal would respond. Four beaked whales were tagged for baseline data, and two tagged whales were exposed to playbacks of these different sounds using a stationary playback source.

**Figure 4:**
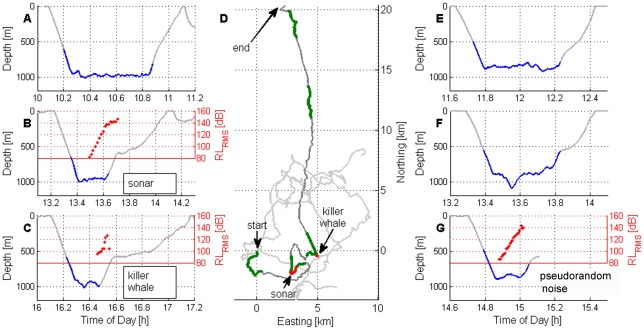
Whale acoustic activity and dive behavior before, during and after sound playback experiments. Deep foraging dives of one beaked whale tagged in 2007 (A–C) and another beaked whale tagged in 2008 (E–G) with the time of each dive indicated on the x-axis. The segment of the dive when the whale was clicking is indicated by coloring the dive profile blue. (A) This first dive after tagging was a pre-exposure dive. (B) This second dive after tagging involved playback of the MFA sonar stimulus, with slowly increasing level of playback. The red dots mark the received sound pressure level of the playback signal as recorded on the whale (in dBrms re 1 µPa averaged over a 200 msec window). (C) This third dive involved playback of killer whale (ORCA) sounds. The received level of the ORCA playback signal indicated by the red dots is the third octave band with the most energy averaged over a 200 msec. (D) The horizontal component of the motion of the tagged whale exposed to playbacks in 2007 is plotted in the dark gray and colored lines. Deep foraging dives are marked in green, and playback sound exposure is indicated in red. Tracks of three other beaked whales tagged during baseline conditions when no sonar was transmitting are shown in light gray for comparison. All tracks are presented with respect to the same arbitrary start position at (0, 0). Note that none of the baseline tracks contain segments as linear as the avoidance response of the whale after playback of killer whale sounds. (E–F) These first two dives after tagging in 2008 were pre-exposure dives. (G) The third dive after tagging in 2008 involved playback of the PRN sound stimulus, with slowly increasing level of playback. The red dots mark the increasing received sound pressure level of the playback signal (in dBrms re 1 µPa averaged over a 200 msec window).

**Figure 5:**
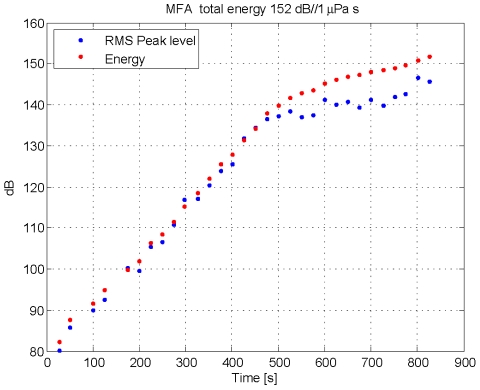
Exposure to MFA sonar pings during the playback in 2007. The blue dots indicate the highest sound pressure level (SPL) in 200 msec windows of each MFA ping in dB re 1 µPa. The energy within the whole ping integrated over 2 s in dB re 1 µPa^2^ s is integrated over all earlier pings to provide a cumulative Sound Exposure Level (SEL) indicated by the red dots. The overall SEL for the entire exposure is calculated by integrating energy of all the pings and is 152 dB re 1 µPa^2^ s.

Fig. 4A shows the depth profile of the first dive after tagging for an adult female Blainville's beaked whale that was tagged in 2007. This first dive was considered a pre-exposure control dive. During the second dive (Fig. 4B) the subject was exposed to playback of a simulated 1.4 s MFA sonar signal repeated every 25 s. The level of each subsequent transmission was increased, resulting in a received sound pressure level (SPL) at the whale that varied from inaudible to 147 dB (all SPL levels RMS re 1 µPa within the loudest 1/3^rd^-octave band; 200 ms integration window), (Fig. 5). The whale stopped clicking after 9 minutes when the received level reached 138 dB SPL (Fig. 4B), or a cumulative sound exposure level (SEL) value of 142 dB re 1 µPa^2^-s (Fig. 5). The simulated sonar transmissions were stopped <5 minutes after the whale stopped clicking. Once the whale stopped clicking, she ascended slowly, moving away from the sound source. Integrating the energy from all the pings led to a cumulative SEL value of 152 dB re 1 µPa^2^-s (Fig. 5). The whale surfaced and remained in the area for approximately 2 h before making a third foraging dive.

During the third dive (Fig. 4C) the whale was exposed to recorded killer whale calls played back in the 2–5 kHz frequency band. The broadcast level was increased slowly, resulting in received levels that ranged from below ambient noise up to a maximum of 126 dB SPL. The whale stopped clicking about 1 min after the received level of the killer whale sound reached 98 dB SPL, just above the background noise level at the whale. The whale then made the longest and slowest ascent out of 32 total dives for which ascent was recorded from 6 whales tagged at this site. After surfacing, the whale continued to swim away from the playback location for 10 h (Fig. 4D).

**Figure 6:**
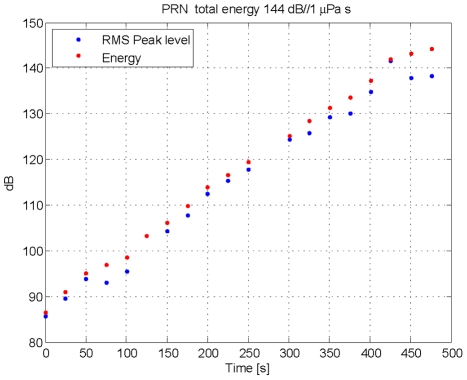
Exposure to PRN pings during the playback in 2008. The blue dots indicate the highest sound pressure level (SPL) in 200 msec windows of each PRN ping in dB re 1 µPa. The energy within the whole ping integrated over 2 s in dB re 1 µPa^2^ s is integrated over all earlier pings to provide a cumulative Sound Exposure Level (SEL) indicated by the red dots. The overall SEL for the entire exposure is calculated by integrating energy of all the pings and is 144 dB re 1 µPa^2^ s.

In 2008 a tagged adult male Blainville's beaked whale in a group of two was allowed to make two pre-exposure dives (Fig. 4 E–F) before being exposed to a PRN signal soon after it started a third deep foraging dive at a horizontal range of about 700 m from the source ship (Fig. 4G). The received level at the whale ranged from inaudible to 142 dB SPL (144 dB cumulative SEL, Fig. 6). The whale stopped clicking <2 minutes after exposure to the last transmission and ascended slowly to approximately 600 m. The whale appeared to stop at this depth, at which time the tag unexpectedly released from the whale. Two beaked whales were resighted on the surface 2.4 km away from the source ship suggesting that the whales made a long, slow ascent moving away from the sound source.

**Figure 7:**
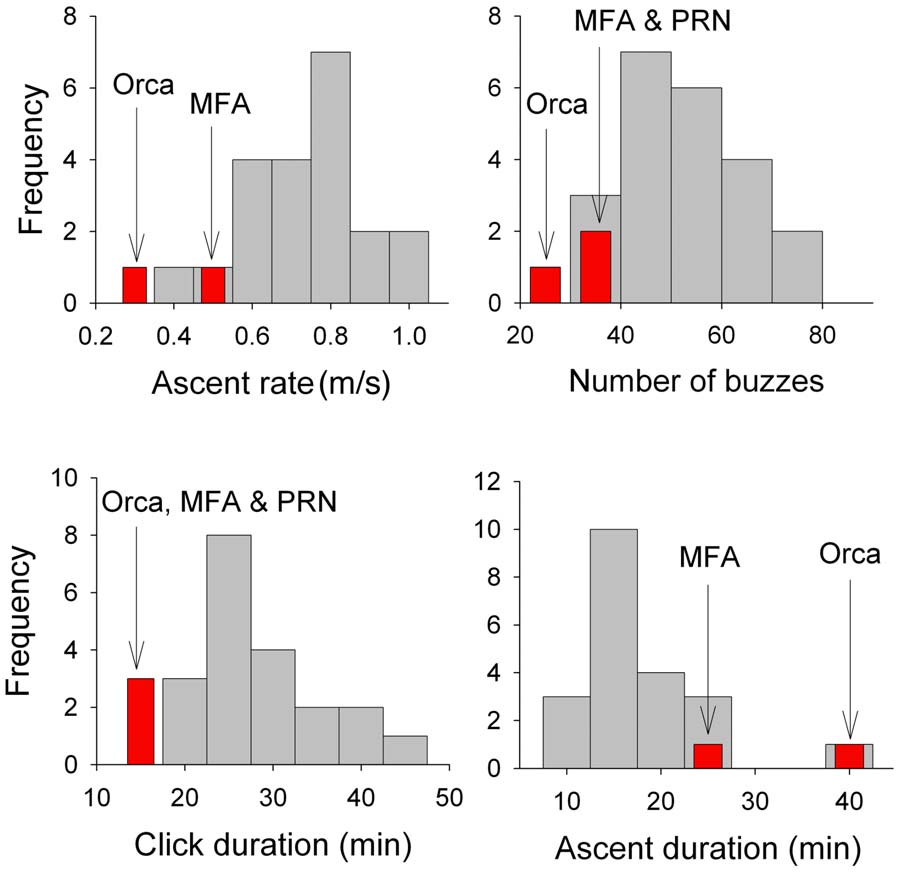
Histograms of the four significant response parameters from the beaked whale playbacks. Exposure dives are indicated in red and baseline dives in gray.

We performed a statistical analysis of dive parameters comparing the 3 exposure dives to baseline dives. Most dive data were available for 33 dives from 6 individuals; all were baseline except for 3 dives of the 2 individuals exposed to acoustic playback. After accounting for the effects of differences between individuals and sex of the 2 playback and 4 baseline whales, foraging and ascent behaviors were significantly affected by the playbacks (Table 3). The playbacks resulted in a reduction in attempts to capture prey (judged by the number of buzzes), shorter foraging durations (judged by the production of clicks), reduced ascent rate, and increased ascent duration compared to the baseline foraging dives recorded from this species in the same location without playback. Dive variables that represented events in advance of playbacks (descent rate, duration and interval before the dive) did not differ between baseline and playback dives, but those occurring during or after playbacks (duration of clicking, number of buzzes, ascent rate, duration of ascent and interval after the dive) were affected. Figure 7 plots the histograms of the values of exposure and baseline dives for all four of these parameters. The values for exposure dives, marked in red, are obvious outliers.

**Table 3:** Results of mixed effects models examining the effects of sound playback on dive variables in Blainville's beaked whales.

Dependent variable	*Df*	Playback		Individual		Sex	
		F	*P*	F	*P*	F	*P*
Pre-dive interval	11	0.92	0.358	0.49	0.699	2.90	0.117
Descent rate	18	0.79	0.386	1.54	0.233	9.79	0.006^***^
Descent duration	18	1.73	0.206	2.75	0.060	11.56	0.003^***^
Duration of clicking	18	14.02	0.001^***^	3.65	0.024^*^	4.95	0.039^*^
No. Buzzes	18	10.81	0.004^***^	1.01	0.429	0.56	0.466
Buzz rate	18	0.05	0.820	0.66	0.629	0.58	0.455
Dive depth	27	1.90	0.180	5.46	0.002^**^	2.31	0.140
Ascent rate	17	10.41	0.005^***^	1.94	0.150	2.13	0.162
Duration of silent ascent	17	11.50	0.004^***^	5.12	0.068	9.03	0.080
Dive duration	26	0.02	0.891	8.25	0.001^***^	16.49	0.004^***^
Post-dive interval	11	5.88	0.034^*^	4.70	0.024^*^	4.10	0.067

## Discussion

Two complementary methods of observing beaked whales exposed to anthropogenic noise during foraging dives documented similar responses within a narrow exposure range that is well below the levels used by regulators in the US as criteria for behavioral disruption in cetaceans [6], [24]. For example, the risk function used to assess probability of behavioral harassment of cetaceans from sonar assumes a very low risk of harassment at the exposure levels near 140 dB, levels at which we observed disruption of foraging behavior [24]. Acoustic monitoring of actual sonar exercises indicates that some beaked whales were exposed to levels >140 dB without ceasing clicking during the sonar exposure, but the gap in distribution seen in Fig. 1B suggests that most whales stopped foraging and moved away to distances of more than 10 km, judging from the 4 km separation between hydrophones. Table 2 shows that this distance is associated with a range of exposure levels, but that these levels are similar to those associated with a reaction in the playback experiments, around 140 dB (Table 2).

The controlled exposure experiments showed that tagged whales responded by silencing to the PRN signal at 142 dB SPL and to the sonar signal at 138 dB SPL. This is higher than the 98 dB SPL level at which a tagged whale responded to killer whale playback, but is similar to the 136 dB SPL previously reported for ship propulsion noise that caused a Cuvier's beaked whale to cease clicking and break off a foraging dive in the Mediterranean Sea [25]. The sample size of the controlled exposure experiments is too small to allow firm conclusions about differential reactions to different stimuli without more replications, but our combined results suggest that the behavior of beaked whales can be disrupted by exposure to anthropogenic sounds at levels near 140 dB, with the response to killer whale sounds occurring at a much lower level of 98 dB.

There is no evidence that beaked whales at AUTEC have stranded during periods when naval mid-frequency active (MFA) sonar is being used. However, our data suggest that beaked whales move tens of kilometers away from sonar exercises there. The avoidance responses reported here reduce exposure to sonar, but if beaked whales move out of their normal deep water habitat into shallow water, this could increase their risk of stranding. Our playback experiments also demonstrated a clear reaction to sonar, involving premature cessation of foraging dives, coupled with a prolonged ascent. A similar response to playback of killer whale sounds was followed by a prolonged avoidance response over tens of kilometers, similar to the scale of avoidance seen for sonar exercises. The tagged whales responded to the sonar exercise and to the killer whale playback not with panicked flight, but with well oriented swimming to the north, which is the only deep water exit from the Tongue of the Ocean. As long as regulators are willing to use the disruption of behavior we observed as a signpost for risk, then we have succeeded in developing an experimental protocol that appears to be safe for the subjects and can be used to establish the acoustic exposure associated with onset of risk.

The small sample size of controlled exposure experiments reported here is too small to make firm conclusions about differential responses of beaked whales to different stimuli, but given this caveat, we do wish to discuss the results in terms of the hypotheses mentioned above. The hypothesis that beaked whales may treat MFA sonar as indicating the possible presence of a killer whale predicts similar responses at similar exposure levels for MFA and killer whale stimuli. This was not observed in our experiment, where much lower levels of killer whale sounds elicited a much more prolonged response than was observed for the MFA sonar playback. Instead, the limited evidence available suggests that beaked whales respond to shipping noise [25] and the MFA and PRN stimuli at similar exposure levels. It is not possible from this single experiment to determine whether this heightened sensitivity and prolonged response were caused by the killer whale stimulus alone, or whether the previous sonar playback to the same whale just hours earlier may have had an influence. If we assume that the greater sensitivity to killer whale sounds than to anthropogenic sounds is caused by recognition that the stimulus is associated with a predator, then our results do not support the interpretation that beaked whales react to MFA sonars at similar exposure levels as they do for potentially lethal predators [26].

The killer whale playback was designed to test how beaked whales respond to predators, and the prolonged avoidance response observed soon after the signal exceeded ambient noise is consistent with anti-predator behavior. The beaked whale response to short-term playbacks of MFA and PRN had much shorter durations than the response to playback of killer whale sounds, but beaked whales in our observational studies did show similar scales of avoidance response to actual sonar exercises as they did to the killer whale playback. This suggests that even though the reaction to short exposures of low-level sonar and band-limited noise were limited to relatively small scales in time and space, beaked whales may invoke an avoidance response similar to anti-predator responses in situations where human activities produce intense sounds for hours. More experiments and observations are required to test whether beaked whales tend to react to most anthropogenic stimuli at levels near 140 dB SPL, and whether they react more strongly at lower exposures of killer whale sounds.

These data are consistent with the conclusion that, similar to harbor porpoises [6], beaked whales are particularly sensitive in terms of behavioral responses to acoustic exposure. In the US, regulators have a separate exposure criterion for harbor porpoise than other cetaceans. Regulators predict that any exposure above 120 dB SPL will disturb behavior in porpoises, while a variety of higher criteria are used for other species [6]. Our results support a similar criterion of about 140 dB SPL for beaked whale exposure to mid-frequency sounds. However, more data from beaked whales are required to finalize a dose:response function, and analyses of similar experiments with different species are required to support the interpretation that other species may be less sensitive than beaked whales. Our results do support a lower acoustic threshold of disturbance for beaked whales than is currently applied in the US.

## Materials and Methods

### Ethics Statement

The research was conducted under permits for marine mammal research issued by the U.S. National Marine Fisheries Service to John Boreman (Permit #1121-1900) and to Peter Tyack (Permit #981-1578), and issued by the Government of the Bahamas to the Bahamas Marine Mammal Research Organisation (Bahamas permit #01/09) and Ian Boyd (Bahamas permit #02/07 and #02/08). This study was carried out in strict accordance with the US Animal Welfare Act following the relevant recommendations of the Guide for the Care and Use of Laboratory Animals of the National Institutes of Health. The experimental research was approved by the WHOI and BMMRO Institutional Animal Care and Use Committees and the Animal Welfare and Ethics Committee of the University of St Andrews.

### Field Site

The field site selected for this project was in the Tongue of the Ocean, a basin of deep water to the east of Andros Island in the Bahamas which is surrounded by islands and sand banks. The only deep water entrance to the basin is to the north. Three different species of beaked whale are sighted in Bahamian waters including the Tongue of the Ocean [27]. Blainville's beaked whales (*Mesoplodon densirostris*) are the most common, but Cuvier's beaked whale (*Ziphius cavirostris*), and Gervais' beaked whale, (*Mesoplodon europaeus*), have also been sighted there [28]. This site was selected not only because of the presence of beaked whales but also because of the demonstrated capability to detect and locate beaked whales acoustically by listening to an array of hydrophones installed by the US Navy as part of its Atlantic Undersea Test and Evaluation Center (AUTEC), which is an underwater acoustic range [17].

The US Navy installed the AUTEC array of hydrophones to track pingers attached to different vessels, and to monitor military exercises, but the hydrophones have also been used to detect and locate marine mammals, including beaked whales [17]. The echolocation clicks of the three beaked whale species present on the range are sufficiently different at this site to be discriminated [28]. In order to validate the ability of acoustic monitors to identify the clicks of Blainville's beaked whales, visual observers were sent to where clicks were heard. On all occasions (n = 12) when whales were sighted soon after clicks stopped being heard, they were identified as Blainville's beaked whales, confirming the species identification of animals detected using acoustic monitoring [17].

The resighting rate of Blainville's beaked whales identified from photographs of natural markings indicates a relatively high rate of resight, but there has also been a consistent discovery of new animals over several years [23], [27]. These results suggest that at least some of the whales are resident, but that other individuals continue to enter or pass through the area. Several times a month, the AUTEC range hosts naval activities that make sounds including the propulsion noise of ships, acoustic pingers and a variety of sonars, but currently exercises with several ships operating mid-frequency sonars are only conducted about twice a year at AUTEC. Any resident animals would routinely hear anthropogenic sounds from naval activities on the range, but the continued resighting of new animals raises the possibility that relatively naïve animals may be exposed to intense naval sounds at AUTEC.

### Passive Acoustic Monitoring System for AUTEC hydrophone arrays

The AUTEC range has 82 hydrophones that are mounted on the seafloor at depths of ≤2000 m and are cabled back to a building on shore. These hydrophones have a high enough upper frequency and close enough spacing to be suitable for tracking echolocation clicks of Blainville's beaked whales, which have a center frequency of about 40 kHz [15]. Signals from each of the AUTEC hydrophones can be recorded for later analysis, but are also displayed for real-time monitoring that can be used to help direct research vessels to the location of the whales [17] and also to monitor positions and vocal behavior of whales before, during, and after naval sonar exercises. Acoustic monitoring and tag data have shown that Blainville's and Cuvier's beaked whales make foraging dives about once every two hours [11], [17]. The whales produce echolocation clicks for about 30 min while foraging at depth during each foraging dive. The source level of Cuvier's beaked whale is about 214 dB re 1 µPa at 1 m [29]; Blainville's beaked whales are somewhat smaller than Cuvier's, and are thought to have a somewhat lower source level, perhaps 200–210 dB re 1 µPa at 1 m. The echolocation clicks of Blainville's beaked whales have a relatively narrow beamwidth, probably comparable to the 6 degree −3 dB beamwidth of Cuvier's beaked whales [29], [30]. The range hydrophones tend to detect short series of echolocation clicks as a whale scans its beam past a hydrophone, although longer series can be recorded when the whales are close enough for off-axis clicks to be detected. Beaked whale clicks can be detected on AUTEC hydrophones at ranges of up to 6500 m, usually when the whale is pointing within 30 degrees of the hydrophone [30]. The typical separation of hydrophones is ∼4 km, so at least some clicks should be recorded from every deep foraging dive. When a group of beaked whales starts echolocating during a deep foraging dive, these sequences of clicks are detected off and on at a few neighboring hydrophones for periods of typically tens of minutes [17]. We defined a group clicking period (GCP) as the time when sequences of clicks corresponding to synchronized foraging dives from one group were detected on a cluster of nearby hydrophones. The start of a GCP was considered to be the occurrence of five or more distinct clicks typical of Blainville's beaked whale (known as a “click train”) within a 30-second time interval. We selected detection of 5 clicks within a 30 second window as providing a good compromise between selecting the loudest clicks in a scan while still providing enough clicks to include inter-click-intervals for classifying the clicks. The end of the vocal period was considered to occur at the end of the last distinct click train followed by more than 3 minutes without clicks on the cluster of hydrophones that had been active. Several different groups of beaked whales can be detected on the range simultaneously, with their clicks being detected by different groups of hydrophones at different locations on the range [17].

### Acoustic Recording Tag

An archival tag (“DTag”) that records data to flash memory was used to sample acoustic data from two hydrophones at a rate of 192 kHz, and to record at a sampling rate of 50 Hz the signals from a thermistor, a pressure sensor for depth, and three-axis magnetometers and accelerometers [22]. Acoustic calibration of the tags was carried out at the Naval Undersea Warfare Center in Newport RI at pressures of up to 5.5 MPa (800 psi, equivalent to a hydrostatic pressure of about 550 m depth) showing that pressure had little effect on hydrophone sensitivity. The acoustic data from the tag were compared to records of detections on the range hydrophones. For clicks that could be detected on several hydrophones, the location of the clicking whale could be calculated based upon the time of travel from the whale to each hydrophone [30]. For georeferencing the track of the tagged whale, these calculated locations were augmented where possible by visual observations of the surfacing tagged whale. The tag includes a VHF radio transmitter that facilitates tracking and resighting of the tagged whale. The tags were deployed for up to 18 hours on each whale using a suction cup attachment [22]. Tags were released after a preset time if they had not already released incidentally due to the movement of the animal or interaction with others.

### Satellite tagging a *Mesoplodon densirostris* near AUTEC, May 2009

A satellite tag was deployed on an adult male Blainville's beaked whale on 7 May 2009 within the AUTEC range in Tongue of the Ocean, Bahamas. The tag was a satellite “dart-tag” (SPOT5 model, Wildlife Computers, Redmond, WA), with a location-only satellite transmitter [21]. This small tag was held on the external surface of the whale at the base of the dorsal fin by two barbed titanium posts that threaded into the tag and were designed to penetrate to a depth of 4.5 cm. The tag was deployed from approximately 10 m using a rifle powered by a .38 caliber blank charge to project the tag on the end of a crossbow bolt, which fell away on contact with the whale. The tag was scheduled to transmit for six 2-hour periods each calendar day, and transmissions from the tag were recorded and processed to estimate animal locations using the ARGOS system (CLS America, Largo, MD).

### Method for Filtering Location Data from the Satellite Tag

Locations estimated from the satellite tag were filtered using the algorithm described in [31] with a maximum swim speed of 3 m s^−1^. Ninety-nine filtered location data points covered the time period between 7 May 2009 and 30 May 2009, and were classed as “pre-exposure”, “exposure” or “post-exposure” depending on their temporal overlap with the Submarine Commander's Course, a multi-ship naval sonar exercise which occurred between 10:00 (GMT) on 14 May 2009 and 10:00 (GMT) on 17 May 2009. The distance was calculated between each location and the approximate center of the AUTEC range (24.5 N, 77.5 W), which was roughly the center of sonar activity.

### Sound stimuli used for playback experiments

Three different sound stimuli were used in the playback experiments to two Blainville's beaked whales at AUTEC: a mid-frequency naval sonar signal (MFA) with both constant frequency and frequency modulated tonal components in the 3–4 kHz band, a pseudorandom noise signal (PRN) with overall bandwidth and timing similar to MFA, and killer whale (ORCA) sounds. The MFA signal transmitted in 2007 was designed to simulate an actual waveform transmitted by the US Navy AN/SQS53-C tactical MFA sonar system [32]. The MFA signal was made up of a 0.5 sec linear frequency modulated upsweep from 3.2 to 3.3 kHz, followed by a 0.5 sec constant frequency tone of 3.43 kHz, followed by a 0.1 sec silent interval, followed by a 0.3 sec constant frequency tone of 3.75 kHz. This signal lasted for an overall duration of 1.4 sec and was repeated every 25 sec. The PRN signal transmitted in 2008 was then designed to have the same overall frequency bandwidth and timing as the MFA signal. The PRN stimulus was made up of a 1 sec signal of noise in the 3.2 to 3.75 kHz frequency band, followed by a 0.1 sec silent interval, followed by a 0.3 sec signal of noise in the 3.2 to 3.75 kHz frequency band. Just like the MFA stimulus, this PRN signal lasted for an overall duration of 1.4 sec and was repeated every 25 sec. The killer whale sounds transmitted in 2007 were a 10-minute segment of recordings from wild marine mammal eating (transient) killer whales (*Orcinus orca*) recorded in Southeast Alaska in the North Pacific by Volker Deecke (University of St. Andrews). Killer whales have been recorded in Bahamian waters by the Bahamas Marine Mammal Research Organisation, but these recordings were not long enough or of sufficient signal to noise ratio to use as stimuli. Harbor seals react to the calls of strange killer whales as a predator [33], and this is typical for reactions to predator calls, reducing concern about using killer whale stimuli from areas other than the Bahamas.

### Sound sources used for playback

The MFA, PRN, and ORCA sounds were transmitted through sound sources designed to broadcast in the 2–5 kHz frequency band. This 2–5 kHz frequency band was selected to cover the frequencies of the MFA stimuli, but had a narrower bandwidth than the sounds produced by killer whales, which include energy below 2 kHz and above 5 kHz [34]. This limitation to the 2–5 kHz frequency band required band-pass filtering of the killer whale waveform to match the 2–5 kHz pass band of the sound transducer. The loss of higher frequencies of the killer whale calls was similar to the frequency dependent attenuation of high frequency calls in seawater [35], leading the playback stimulus to have some acoustic features as if it came from a more distant, higher bandwidth source. This lack of full bandwidth means that the stimulus should probably be viewed as an attenuated killer whale stimulus, especially for animals such as beaked whales, whose best hearing is well above 5 kHz [13], [14].

The acoustic sources used for sound playback in 2007 and 2008 were designed to be deployed from a stationary ship. The sound source used for the 2007 experiment was a single flextensional transducer [36] custom built for the Naval Undersea Warfare Center in Newport, Rhode Island. The sound source used for the 2008 experiment was made up of four Model 1024 free-flooded ring transducers, made by the International Transducer Corporation (Santa Barbara CA). Each transducer was driven with a separate power amplifier. Both sources were designed to transmit in the 2–5 kHz frequency band at a source level of up to 212–214 dB re 1 µPa at 1 m (RMS sound pressure level or SPL), but were not operated beyond the maximum source level of 212 dB re 1 µPa at 1 m used in these experiments. The sound sources were deployed at a depth of 45 m during 2007 and 66 m during 2008 for transmissions to beaked whales during deep foraging dives. The directivity pattern and actual source level for each source were measured at AUTEC before the sound playback experiments. Both sources were somewhat directional, placing more of their output acoustic energy into angles relatively near to the horizontal plane of the source.

For each playback to a beaked whale, the source ship was maneuvered to a position about 1 km from where the whale had begun a deep foraging dive. Sound source transmissions were started only after beaked whale clicks, indicating the start of a deep foraging dive, were heard on the range hydrophones. For each sequence of transmissions, a ramp up was performed, starting at a source level of 152 dB re 1 µPa at 1 m in 2007 and 160 dB re 1 µPa at 1 m in 2008, increasing 3 dB every 25 seconds and continuing until beaked whale clicks were no longer heard or the maximum source level of 212 dB re 1 µPa at 1 m in 2007 and 211 dB re 1 µPa at 1 m in 2008 was achieved. The longest exposure involved 9 min of ramp up followed by 16 pings of the MFA stimulus at the maximum source level over a period of 6.3 min. The 2007 ORCA transmission started at an initial source level of 130–140 dB re 1 µPa at 1 m and was then increased by about 5 dB every 30 seconds reaching a maximum SL of 190–203 dB re 1 µPa at 1 m.

### Calculation of received level of playback stimuli

**Figure 8:**
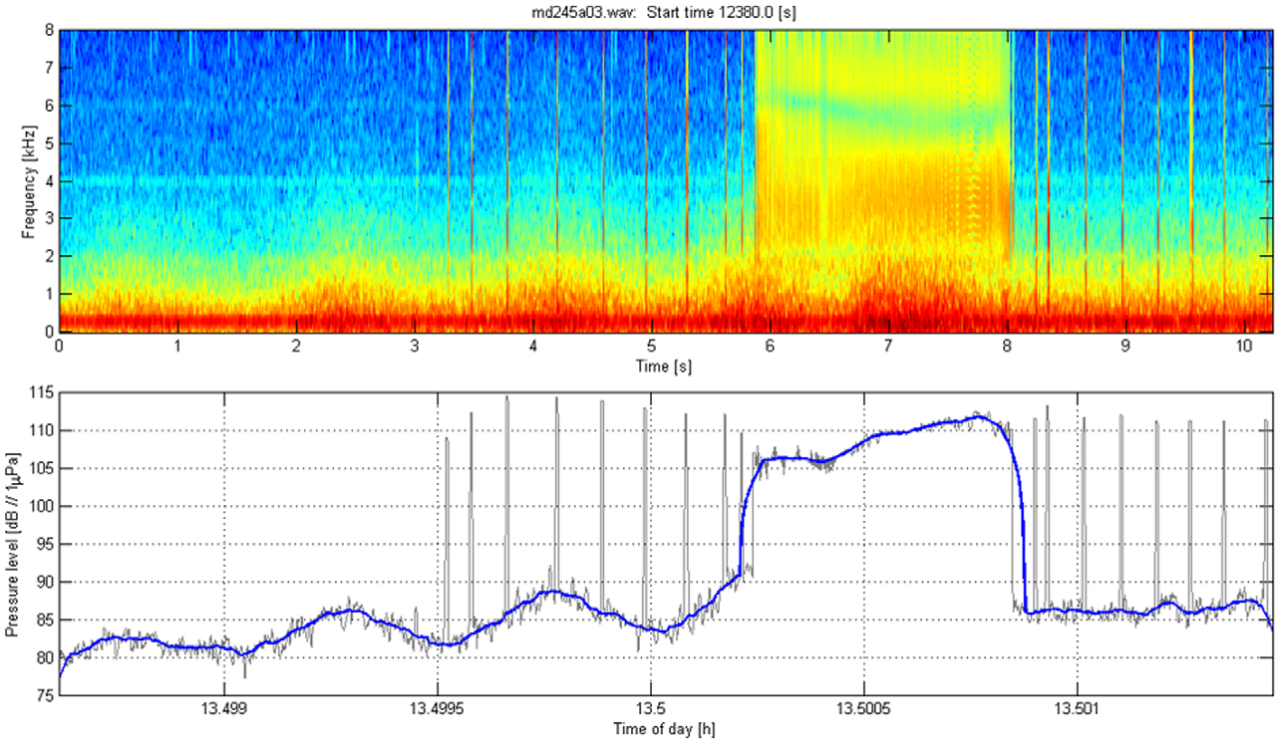
Illustration of the method used to estimate acoustic exposure for beaked whale sounds. Top: Spectrogram of regular echolocation clicks and a buzz from a tagged *Mesoplodon densirostris* at AUTEC. Low-frequency flow noise from swimming motions is also visible below 1 kHz. Bottom: Calculation of RMS sound levels in the 1/3^rd^-octave band in dB re 1 µPa used for the analysis of the MFA and PRN signal (3111–3920 Hz). The thin black line uses a 10 msec window for calculating RMS, the thicker blue line uses a 200 msec window. The algorithm that calculates RMS over the 200 msec window includes a routine that rejects energy from short echolocation clicks.

**Figure 9:**
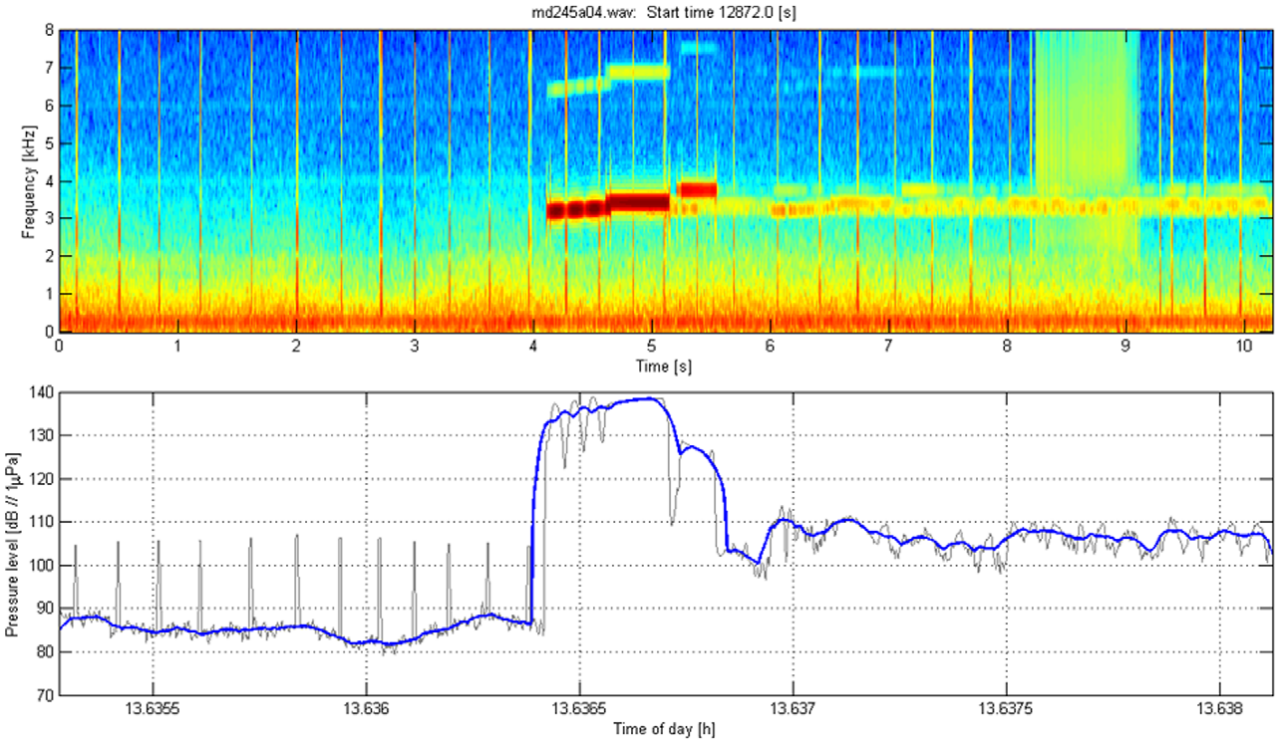
Illustration of the method used to estimate acoustic exposure for MFA sonar sounds. Top: Spectrogram of a mid-frequency sonar signal (MFA) broadcast during sound playback in September 2007 as recorded on a tagged *Mesoplodon densirostris*. The direct arrival of the playback signal occurred at time 4.2–5.5 sec; sound energy at the same frequency later in the spectrogram represents reverberation of this signal arriving from other paths such as reflections from the surface and the seafloor. Low-frequency flow noise from swimming motions is also visible below 1 kHz. Bottom: Calculation of RMS sound levels in dB re 1 µPa in the 1/3^rd^-octave band used for the analysis of the MFA and PRN signal (3111–3920 Hz). The thin black line uses a 10 msec window for calculating RMS, the thicker blue line uses a 200 msec window.

**Figure 10:**
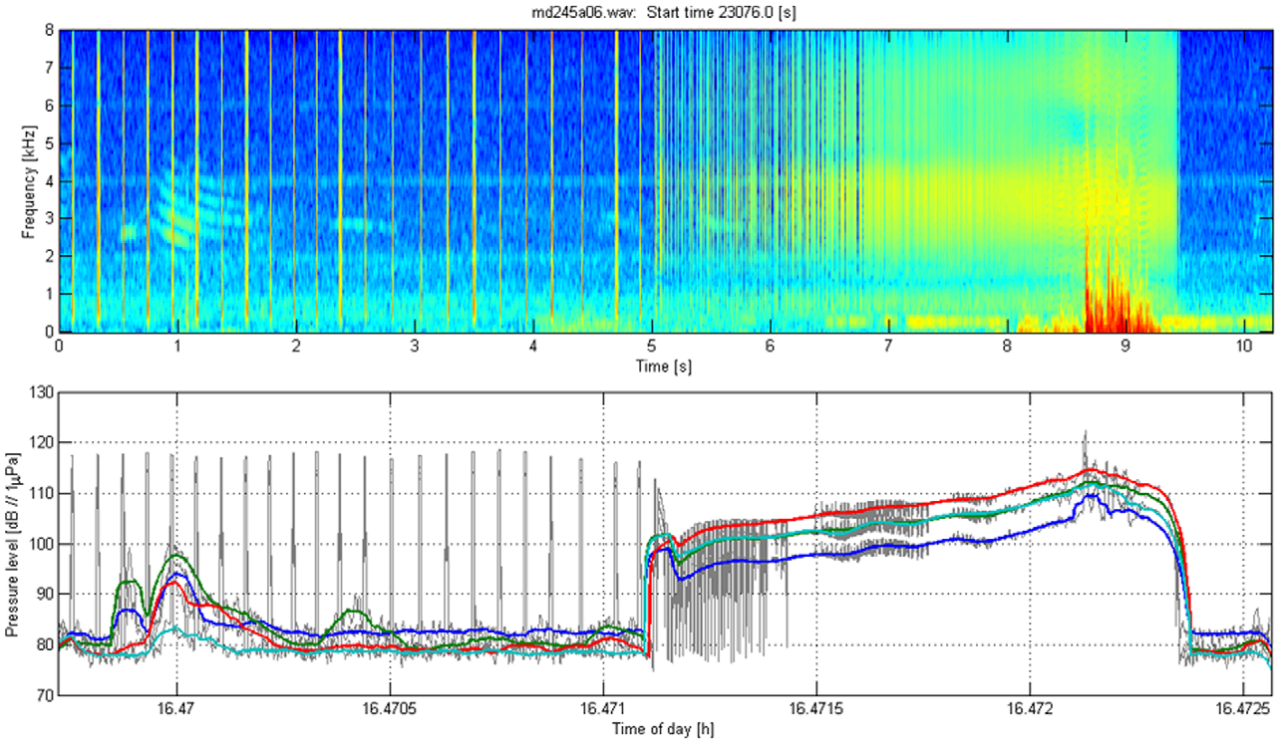
Illustration of the method used to estimate acoustic exposure for killer whale sounds. Top: Spectrogram of a killer whale (*Orcinus orca*) signal broadcast during sound playback in September 2007 as recorded on a tagged *Mesoplodon densirostris*. The main killer whale signal occurred between 0.75–1.5 sec. The tagged whale produced a buzz from 6–9.5 sec. Bottom: Calculation of RMS sound levels in dB re 1 µPa. The thin black line shows the results from using a 10 msec window for calculating RMS, the colored lines indicate RMS energy in 1/3^rd^-octave bands between 2 and 5 kHz over a 200 msec window. The blue line indicates the 1/3^rd^-octave band between 1960–2467 Hz, the green line indicates the 1/3^rd^-octave band between 2467–3111 Hz, the red line indicates the 1/3^rd^-octave band between 3111–3920 Hz, and the cyan line indicates the 1/3^rd^-octave band between 3920–4939.

**Figure 11:**
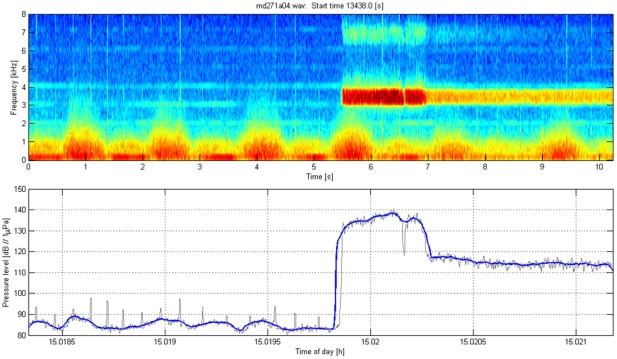
Illustration of the method used to estimate acoustic exposure for pseudo-random noise. Top: Spectrogram of a pseudorandom noise (PRN) signal broadcast during sound playback in September 2008 as recorded on a tagged *Mesoplodon densirostris*. The direct arrival of the playback signal occurred at time 5.5–7 sec; sound energy at the same frequency later in the spectrogram represents reverberation of this signal arriving from other paths such as reflections from the surface and the seafloor. Low-frequency flow noise from swimming motions is also visible below 1 kHz. Bottom: Calculation of RMS sound levels in dB re 1 µPa based on a single 1/3^rd^-octave band (3111–3920 Hz). The thin black line shows the results using a 10 msec window for calculating RMS, the thicker blue line used a 200 msec window.

The acoustic recording tag on each Dtagged whale recorded the playback stimuli at the whale. The acoustic sensors on the tags were calibrated with respect to a reference hydrophone, allowing estimation of the received level of sound at the whale for each playback transmission that was recorded. Figs. 8, 9, 10, 11 illustrate how received level (RL) was estimated for sounds of beaked whales (Fig. 8), MFA sonar (Fig. 9), ORCA (Fig. 10), and PRN (Fig. 11). The bottom panels of Figs. 8, 9, 10, 11 indicate RMS levels calculated over 10 and 200 msec intervals. The algorithm that calculates RMS level over the 200 msec window has a routine that rejects energy from short echolocation clicks. The maximum value of RM sS sound pressure level averaged over 200 msec within a third octave band for each playback signal was selected for results reported in the paper and plotted on Fig. 4. The MFA and PRN signals all had energy within one 1/3^rd^-octave band, and the RMS level was calculated within the 1/3^rd^-octave band that included the bandwidth of the signal, 3111–3920 Hz. The killer whale signal contained energy from 2–5 kHz, across 4 1/3^rd^-octave bands. To cover the whole frequency range of the ORCA broadcast, the analysis used four 1/3^rd^-octave bands with the following five edge frequencies: 1960, 2467, 3111, 3920, and 4939 Hz. The RL used for the ORCA signal was then the maximum level from the 1/3^rd^-octave band represented by the green line at time 16.47 h (RL∼98 dB); the higher levels near 16.4722 h come from a buzz produced by the tagged whale.

### Analysis of energy within each transmission and cumulative Sound Exposure Level

As a first step in calculating the cumulative Sound Exposure Level, the acoustic energy of each playback transmission was calculated over a two second window to include early multipath arrivals. As can be seen in Figs. 9 and 11, this window does not include all of the reverberations of the signal, but it does include most of the energy while minimizing the effect of noise within the transmit band. For each ping of MFA (Fig. 9) and PRN (Fig. 11, the energy values (red dots in Figs. 5 and 6) were slightly lower than the maximum SPL within a 200 msec window. The cumulative Sound Exposure Level [37], given in the title of each of Figs. 5 and 6, was calculated by integrating the energy of all the pings, and is dominated by the few last, loudest pings.

### Statistical Analyses

#### Monte Carlo analysis of effects of sonar activity on group clicking periods

A Monte Carlo simulation was used to carry out a two-tailed test for the effects of sonar operations on the frequency of beaked whale group clicking periods observed during each phase of sonar exposure, before, during, recovery, and in 2008, post-recovery. This involved the creation of 1 million randomized time series of group clicking periods across the total duration of observation in each of 2007 and 2008. The observed time series were compared with these random time series to assess five hypotheses:

- H_1_: The rate of group clicking periods before sonar operations began was not significantly different from the rate during sonar operations;
- H_2_: The rate of group clicking periods while sonars were in operation was not significantly different from the rate after sonar operations had ended, during the recovery phase;
- H_3_: The rate of group clicking periods during sonar operations was not significantly different from the rate during the post-recovery period;
- H_4_: The rate of group clicking periods before sonar operations began was not significantly different from the rate during the recovery period.
- H_5_: The rate of group clicking periods before sonar operations began was not significantly different from the rate during the post-recovery period.

The results of the analysis are summarized in Fig. 2.

#### Parameters used to define responses of Dtagged whales to experimental playback

The response parameters used to analyze responses of Dtagged beaked whales to experimental playbacks were defined for each deep foraging dive of each Dtagged whale under baseline or exposure conditions. The responses include the following: pre-dive interval, descent rate, descent duration, duration of clicking, number of buzzes, buzz rate, duration of silent ascent, ascent rate, dive depth, dive duration, and post-dive interval. These parameters were defined following [11] based upon the audio and depth data from the tag. All of the tag acoustic data were audited by at least one person who scrolled through spectrograms such as those illustrated in Fig. 8, and listened to the audio data where this was useful for interpreting the spectrogram. The descent phase of a deep foraging dive is considered to start the last time the whale leaves the surface before performing a deep dive in which it produces echolocation clicks, and to end when the whale starts echolocating. The descent duration is the time between these two events and the descent rate is calculated from the depth at which the whale started clicking divided by the descent duration. The duration of clicking is the time from the first to last click of the dive. The ascent phase of the dive starts at the last regular click and ends when the whale next reaches the surface. The ascent duration is the time between these two events and the ascent rate is calculated from the depth at which the whale stopped clicking divided by the ascent duration. As Blainville's beaked whales echolocate to forage, they occasionally switch from regular search clicks to a buzz, which involves an increase of the repetition rate of clicking and use of a different kind of click [15]. These buzzes are thought to represent attempts to capture prey [12]. The transition from search clicks to an echolocation buzz is illustrated in Fig. 8. Along with the start and stop of regular echolocation clicks, the time for every buzz was noted in an audit of the tag audio data. The number of buzzes is defined as the total number of buzzes identified to the tagged whale during each dive, and the buzz rate is this number divided by the duration of clicking. The dive depth is the maximum depth recorded during each dive, and the dive duration is the time from when the whale left the surface to when it next surfaced at the end of the dive.

#### Background on statistical analysis underlying Table 3 on effects of playback, individual, and sex on dive parameters in Mesoplodon densirostris at AUTEC

Dive depths and durations were available for 33 dives from 6 individuals. All other variables were measured in 25 dives from 6 individuals except for the intervals between deep dives where a sample size of 17 intervals was available and for the statistics of the ascent portion of the dive where there was a sample of 32 dives. The tag came off during the ascent portion of the dive when PRN was played back – this is the dive for which ascent data are incomplete. Variables were tested for normality using Shapiro-Wilk, Kolmogorov-Smirnov, Anderson-Darling and Cramér-von Mises tests. Duration of clicking, number of buzzes, and descent duration were all found to be normally distributed. Dive depth and dive duration were transformed using a Box-Cox transformation with λ = −2. Descent rate, ascent rate and duration of silent ascent were log transformed. All transformed variables were normally distributed. Akaike's Information Criterion was used to determine the most parsimonious model in each case. The inclusion of all three independent fixed effects (playback, sex, and individual) was found to provide the best fit for most cases. In models of ascent rate and buzz rate, sex did not have a significant effect, but exclusion of sex as a fixed effect produced only slight improvement in the model fit. The models showed that there were significant differences in dive depth and dive duration, duration of clicking and post-dive interval between individual whales, but not in any other dive characteristic. Only two of the 6 individuals were male but, in this case, sex appears to have had a significant effect upon duration of clicking, descent rate, descent duration and dive duration.
